# N-gram analysis of 970 microbial organisms reveals presence of biological language models

**DOI:** 10.1186/1471-2105-12-12

**Published:** 2011-01-10

**Authors:** Hatice Ulku Osmanbeyoglu, Madhavi K Ganapathiraju

**Affiliations:** 1 Department of Biomedical Informatics, University of Pittsburgh, 5150 Center Ave, Suite 301, Pittsburgh, PA, 15232, USA

## Abstract

**Background:**

It has been suggested previously that genome and proteome sequences show characteristics typical of natural-language texts such as "signature-style" word usage indicative of authors or topics, and that the algorithms originally developed for natural language processing may therefore be applied to genome sequences to draw biologically relevant conclusions. Following this approach of 'biological language modeling', statistical n-gram analysis has been applied for comparative analysis of whole proteome sequences of 44 organisms. It has been shown that a few particular amino acid n-grams are found in abundance in one organism but occurring very rarely in other organisms, thereby serving as genome signatures. At that time proteomes of only 44 organisms were available, thereby limiting the generalization of this hypothesis. Today nearly 1,000 genome sequences and corresponding translated sequences are available, making it feasible to test the existence of biological language models over the evolutionary tree.

**Results:**

We studied whole proteome sequences of 970 microbial organisms using n-gram frequencies and cross-perplexity employing the Biological Language Modeling Toolkit and Patternix Revelio toolkit. Genus-specific signatures were observed even in a simple unigram distribution. By taking statistical n-gram model of one organism as reference and computing cross-perplexity of all other microbial proteomes with it, cross-perplexity was found to be predictive of branch distance of the phylogenetic tree. For example, a 4-gram model from proteome of *Shigellae flexneri 2a*, which belongs to the *Gammaproteobacteria *class showed a self-perplexity of 15.34 while the cross-perplexity of other organisms was in the range of 15.59 to 29.5 and was proportional to their branching distance in the evolutionary tree from *S. flexneri*. The organisms of this genus, which happen to be pathotypes of *E.coli*, also have the closest perplexity values with *E. coli.*

**Conclusion:**

Whole proteome sequences of microbial organisms have been shown to contain particular n-gram sequences in abundance in one organism but occurring very rarely in other organisms, thereby serving as proteome signatures. Further it has also been shown that perplexity, a statistical measure of similarity of n-gram composition, can be used to predict evolutionary distance within a genus in the phylogenetic tree.

## Background

Microbes are the most diverse organisms on earth. Genomic and proteomic sequences of most major microbes are either already available or soon to be released; these sequences provide an almost overwhelming amount of information about the microbes and their genetic makeup. The first bacterial genome sequence was reported in 1995 [1] and now more than 1,000 genome and proteome sequences of microbes including plant, animal and human pathogens, are available publicly (http://www.ncbi.nlm.nih.gov/genomes/lproks.cgi). With the rapidly increasing availability of whole genome and proteome sequences of microbes, large scale computational recognition and comparison of patterns in biological sequences could be a first step towards discovering and understanding the biology of microbes and their diversity. Understanding their diversity is important to make progress in the field of medicine, public health and agriculture [2], and possibly in exploring alternate energy sources [3]. Currently, the widely accepted method for studying phylogeny (diversity) of microbes is based on a comparison of genes that encode a small subunit RNA (SSU rRNA) [4]. However, as more gene sequences become available, SSU rRNA based grouping has begun to produce results that conflicts with the results from those derived from alternative gene sets [5]. The use of the whole genome/proteome is considered to provide more robust information for grouping of organisms than the information provided by selected gene sets [6]. However, comparison of whole genomes/proteomes may not be feasible for large sets of organisms using multiple sequence alignment (MSA) based methods as only a small portion of genes is shared across all the organisms that are being compared. Orthologous genes comparison (eg. as shown in [7]) which requires correct selection of orthologous genes, protein sequence/structure domains comparison (eg. as shown in [8,9]) which requires the assignment of protein domains at the sequence/structure level, and whole genome/proteome sequences (the pair-wise alignment eg. as shown in [10] or the alignment free eg. as shown in [11]) are the main approaches for inferring whole-genome-based phylogeny of microbial organisms.

In their previous work, Ganapathiraju et al. have suggested that genome or proteome sequences show characteristics typical of natural-language texts, and drawing upon this analogy of biology and language [12] algorithms originally developed for natural language processing may be applied to study biological sequences: topic detection algorithms to secondary or transmembrane structure prediction, statistical n-grams for protein or proteome classification, etc.

N-grams are sequences of 'n' words in a running text. The different n-grams that occur in a document and the frequency of occurrence of each n-gram can be used to characterize the topic of the document or the author-style. N-gram frequencies or more sophisticated statistical models of n-grams are widely used for text processing applications such as information retrieval [13], language identification [14], automatic text categorization [15] and authorship attribution [16]. In a biological context, n-grams can be sequences of amino acids or nucleotides. By employing this analogy between natural language texts and biological sequences, namely by applying 'biological language modeling', whole proteome sequences of microbial organisms have also been shown to contain n-gram genome-signatures [17].

First, Ganapathiraju, et al. [17] compared the n-gram frequencies of 44 different organisms using the simple Markovian uni-gram model (context independent amino acid model). For the proteins of *Aeropyrum pernix*, when the training and the test set were from the same organism, a perplexity of 16.6 was observed, whereas data from other organisms varied from 16.8 to 21.9. This showed that the differences between the 'sublanguages' of the different organisms were automatically detectable with even the simplest language model. They also demonstrated that the modified Zipf-like analysis could reveal specific differences in n-grams (proteome signatures) in different organisms. In other words, specific n-gram sequences were found in abundance in one organism but very rarely in other organisms, thereby serving as the proteome-signature of that organism. Further, it has also been proposed that a statistical model of n-grams (more specifically *perplexity*) of proteome sequences varied from organism to organism. At the time biological language modeling approach was proposed (2002), proteome sequences of only 44 organisms were available, thereby limiting the generalization of this hypothesis.

N-gram based methods also have been successfully applied to biological domain. Karlin et al. introduced a "genomic signature" based on dinucletiode odds ratio (relative abundance) values which appeared to reflect the species-specific properties of DNA modification, replication and repair mechanism [18]. Campbell et al. compared dinucleotide frequencies (genomic signatures) of prokaryote, plasmid, and mitochondrial DNA [19]. They showed that plasmids and their hosts have substantially compatible nucleotide signatures. Mammalian mitochondrial genomes were very similar, and animal and fungal mitochondria were generally moderately similar, but they diverged significantly from plant and protist mitochondria sets. Passel et al. studied genome-specific relative frequencies of dinucleotides of 334 prokaryotic genome sequences [20]. Intrageneric comparisons showed that in general the genomic dissimilarity scores were higher than in intraspecific comparisons. However, genera such as *Bartonella *spp., *Bordetella *spp., *Salmonella *spp. and *Yersinia *spp. had low average intrageneric genomic dissimilarity scores and they suggested that members of these genera might be considered the same species. On the other hand, they observed high genomic dissimilarity values for intraspecific analyses for organisms such as *Prochlorococcus marinus*, *Pseudomonas fluorescens, Buchnera aphidicola *and *Rhodopseudomonas palustris *and they suggested that different strains from the same species might actually represent different species. Recently, Pandit et al. identified the distinctive genomic signature associated with the DNA sequence organization in different HIV-1 subtypes [21].

One of the other earlier applications is protein classification based on n-gram frequencies [22]. Cheng et al. and Daeyaert et al. used n-gram composition of amino acid sequences for protein classification [23,24]. King et al. presented an n-gram-based Bayesian classifier that predicts the localization of a protein sequence [25]. Recently, Maetschke et al. developed an alignment-free and visual approach to analyze sequence relationship of proteins [26]. They used the number of shared n-grams between sequences as a measure of sequence similarity and rearranging the resulting affinity matrix applying a spectral technique. They made use of heat maps of the affinity matrix to identify and visualize clusters of related sequences or outliers and n-gram-based dot plots and conservation profiles to allow detailed analysis of similarities among selected sequences.

N-gram composition based approaches have also been applied to phylogenetic analysis. Stuart et al. used the singular value decomposition of a sparse 4-gram frequency matrix to represent the proteins of organisms uniquely and precisely as vectors in a high-dimensional space [27]. Then, they used vectors of this kind to calculate pair-wise distance values based on the angle between the vectors, and generated phylogenetic trees of mitochondrial genome based on the resulting distance values. Alternatively, Qi et al. developed a method to reconstruct phylogenetic tree based on n-gram frequencies from which random background is subtracted and neighbor joining method is applied [28]. Tomovic et al. also developed classification and unsupervised hierarchical clustering of genome based on n-gram profile similarity measure [29].

Diverse n-gram based methods for identification of compositionally different regions have been devised. For example, Mitic et al. reported genomic island determination via binary classification of islands based on n-gram frequency distribution [30,31]. Rani et al. demonstrated n-gram based promoter prediction where n-grams are used to determine a special bias towards certain combinations of base pairs in the promoter sequences [32].

In language modelling, the most common metrics for assessing n-gram model composition is perplexity [33], which can be interpreted as the (geometric) average branching factor of the language according to the model. Perplexity is a function of both the language as well as that of the model. When considered a function of the model, it measures how good the model is (the better the model, the lower the perplexity). The higher the perplexity, the more branches need to be considered statistically. Perplexity has been used to test performance of language models in a wide range of areas. Speech recognition tasks [33,34], linguistic steganography detection [35], identification of news coverage [6] are some of the examples of the perplexity measure usage. In biological sequence modelling, Buehler et al. [36] used the perplexity metric as a measure of their success in showing that the use of "long distance" features can improve the maximum entropy based model of amino acids sequences.

In this study, we use Zipf-like analysis and the perplexity measure to study the diversity among proteome sequences of microbial organisms as first proposed by Ganapathiraju et al. [17] to address the question of whether or not the sequences in proteins of different organisms are statistically similar or whether organisms may be viewed to possess different languages. Today, with several ongoing genomics efforts, nearly 1,000 microbial genome sequences, and corresponding translated sequences are available, making it feasible to test the existence of biological language models over the evolutionary tree. Here, we extend the previous work [17] with 970 whole microbial proteome sequences and discuss how n-grams truly reveal proteomic signatures and demonstrate how the n-gram statistical language model could be indicative of evolutionary divergence at the genus level.

## Methods

### Dataset

**Figure 1:**
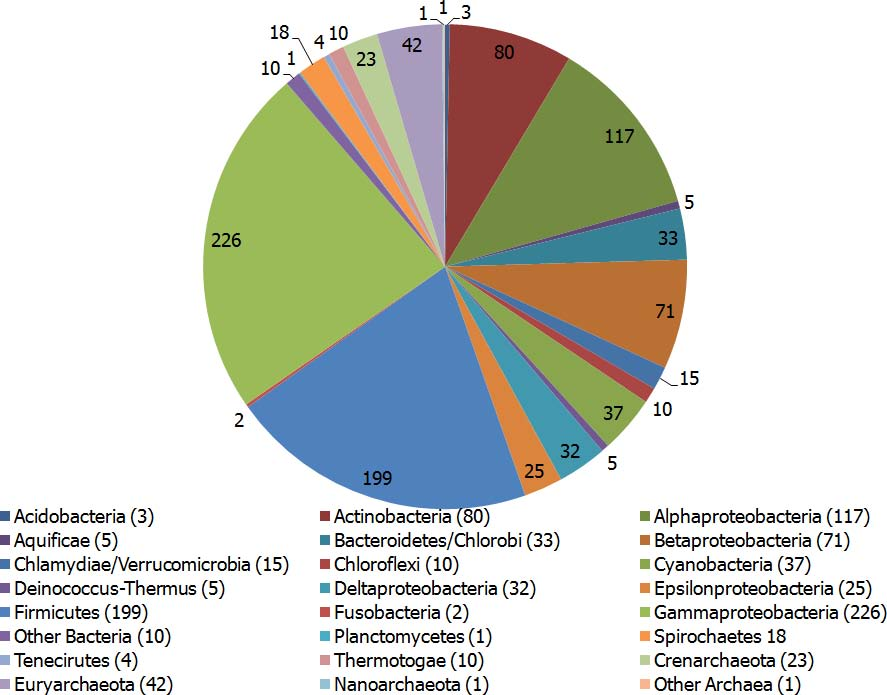
**Dataset**. The pie-chart represents distribution of microbial organisms in the dataset. For proteobacteria which is a very large phylum, the classes within this phylum are labelled. For all other phyla only the phylum name is shown.

Our dataset is comprised of all available translated chromosomal and plasmid amino acid sequences from whole-genome sequences of 970 different microbial organisms downloaded from NCBI (January, 2010). The whole microbial proteomes that belong to bacteria and archaea super kingdoms are 903 and 67, respectively. More details on the distribution of microbial organisms into classes are given in Figure 1.

### N-gram analysis

N-grams are sequences of *n *words. In a biological context, n-grams can be sequences of *n *amino acids or nucleotides. For instance, the sequence "AAANTSDSQKE" has two count of the 2-gram AA, and one count each of the 2-grams AN, NT, TS, SD, DS, SQ, QK and KE. The formal definition of n-grams is given below:

#### Definition 1

Given a sequence of *N *words S = s_1_s_2_...s_N _over the vocabulary A, and *n *a positive integer, an n-gram of the sequence *S *is any subsequence s_i_...s_i+n-1 _of *n *consecutive words[37]. There are N-n+1 such n-grams in *S*. For a vocabulary *A *with |A| distinct words, there are |A|^n ^possible unique n-grams.

### Zipf-like analysis

Zipf's law is based on observations made by the linguist George Kingsley Zipf and states that the most frequent word in any kind of text is expected to be twice as frequent as the second most frequent word, etc. In this study, we used a modified Zipf-like analysis as employed by Ganapathiraju et al. [17] to explore the differences between n-gram usage in different organisms. First, amino acid n-grams of a given length are sorted in descending order by the frequency with which they occur in a reference organism of choice. In all the figures pertaining to this type of analysis, the frequencies of the reference organism are shown in bold line. For comparative analysis, the corresponding frequencies of these n-grams in all other organisms are shown in thin lines. For microbes that are associated with animal hosts, the lines are shown in red and those that are associated with plant hosts are shown in blue.

### Perplexity analysis

In text-processing, for a known corpus and its corresponding language model (for instance, a 4-gram model), how well the language model predicts a new text composed of unseen sentences can be estimated by computing its perplexity [6]. The entropy of its words (*H*) determines the perplexity (*2*^*H*^) of a text. We take the n-grams of the new text, and compute what the probability is of generating that n-gram with respect to the n-gram distribution of the reference text. The lower the perplexity, the better the unseen text fits to the known corpus. When applied to amino acid sequences of whole proteome of organisms, it can reveal how similar a new organism's sequence is to known organisms. This analysis can give us inside into evolutionary relatedness of organisms. The formal definition of perplexity and related terms are given below:

#### Definition 2

Let p(x) be the probability mass function of a random variable X, over a discrete symbol (or alphabet) X: p(X) = P(X = x), x ∈ X

The entropy is the average uncertainty of a single random variable [38]:

(1) $$E(p)=E(X)=\sum_{x\in X}p(x)\log_{2}p(x)$$

#### Definition 3

The cross-entropy between a random variable X with true probability distribution p(x) and probability mass function q (normally a model of p) is given by [38]:

(2) $$E(X,q)=\sum_{x}p(x)\log_{2}q(x)$$

#### Definition 4

In terms of n-gram analysis, perplexity is a measure of the average branching factor and can be used to measure how well an n-gram predicts the next juncture type in the test set. If *N *is the order of the n-gram and *Q *is the number of junctures in the test set, the perplexity *B *can be calculated from the entropy *E *by [38]:

(3) $$B=2^{E}$$

where

(4) $$E=-\frac{1}{Q}\sum_{i=1}p(x)\log_{2}q(j_{i}|j_{i-1},...,j_{i-N+1})$$

With respect to n-grams, perplexity is given for previous *n-1 *letters in a sequence denoting how many different letters can occur in the n^th ^position on an average. For example, given any two letters in the sequence AACCTAACCTAACCTAA CCTAACC..., the third letter can be only one out of 4 possibilities. In other words, perplexity is only 1 in guessing the 3^rd ^letter given two previous letters in the sequence (as opposed to being 4 for a random sequence of nucleotides).

In this study, perplexity is defined by frequencies of n-grams and n-1 grams computed as follows:

For each n-gram denoted as n-gram_j_, its count in both training and test set data are found and denoted as C_train-nj _and C_test-nj_, respectively.

The counts of the (n-1) gram for n-gram_j _(i.e the sequence of the first n-1 characters in n-gram_j_) are also found and denoted as C_train-(n-1)j _and C_test-(n-1)j_

Then the entropy of the test sequence is computed as

(5) $$E=-\frac{1}{N}\sum_{j=1}C_{test-nj}\log_{2}(\frac{C_{train-nj}}{C_{train-(n-1)j}})$$

where j represents the j^th ^n-gram and N is the count of all the n-grams in the sequence.

Perplexity is computed as *2*^*E*^.

### Multinomial Logistic Regression

Multinomial logistic regression (MLR) is used for multi-class classification where the dependent variable is polytomous and independent variables (predictors) are numerical or categorical. The model is generalization of logistic regression where the binary dependent variable is interpreted as occurrence or non-occurrence of a characteristic. It is expressed in the form

(6) $$\log\left(\frac{\mathrm{Pr}}{1-\mathrm{Pr}}\right)=b_{0}+\sum_{i=1}^{n}b_{i}x_{i}$$

where is the intercept and the *b*_*i*_'s denote the unknown logistic regression coefficients of *x*_*i *_parameters (ngram occurences) while *Pr *denotes the probability that the characteristic will occur. The quantity on the left side of the equation is called a *logit*. The model can be generalized in the case where the dependent variables have more than two categories. For possible q categories, q-1 logits are needed to be modelled as

(7) $$\log\left(\frac{\mathrm{Pr}({category}_{j})}{\mathrm{Pr}({category}_{q})}\right)=b_{0}^{\left(j\right)}+\sum_{i=1}^{k}b_{i}^{j}x_{i},j=1,...,q-1$$

As seen from the above equation, one of the categories is used as reference (baseline category). After estimating the coefficients of the model by maximum likelihood model, the probabilities of each one of the categories can be calculated. The final prediction is the category with highest probability [39].

### Suites of tools

Biological Language Modeling Toolkit (BLMT) [40] and Patternix Revelio (under review) are two suites of tools for proteome and genome sequence processing, developed by Ganapathiraju and others. The suites contain tools for computing n-gram frequencies and perplexity, and are designed to use data preprocessing in suffix arrays for efficient comparisons of large scale sequences. All of the computations presented here have been carried out with these two suites of tools.

## Results and Discussion

### Unigram signatures of whole proteomes

**Figure 2:**
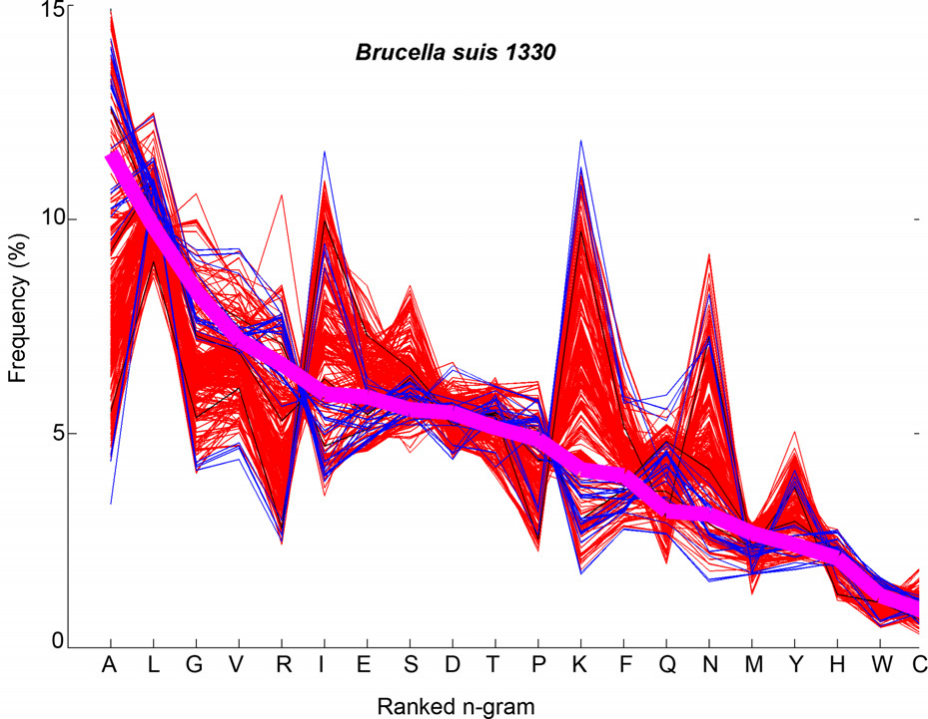
**Comparative Zipf-like analysis for unigrams**. Distribution of amino acid n-grams with n = 1 in *Brucella suis 1330 *in comparison with the distribution of the corresponding amino acids in other organisms is shown. The unigrams on the x-axis are arranged according to their rank in *B. Suis 1330*. Percentage count of n-grams of *Brucella suis 1330 *are plotted in bold magenta line. Corresponding frequencies of unigrams in the other animal pathogens are shown in thin red and plant pathogen in thin blue lines.

We performed the modified Zipf-like analysis to investigate word-usage in whole proteomes of all the 970 microbial organisms in our dataset. In Figure 2, the frequencies of the unigrams of *Brucella suis 1330 *are shown in bold magenta. The x-axis shows the unigrams (amino acids) in descending order of their frequency in *B. suis 1330*. Frequencies of corresponding unigrams in other plant pathogens are shown in thin blue lines and those in animal pathogens are shown in thin red lines. The rank of a specific unigram refers to its position when listed in descending order of frequency. For *B. Suis 1330 *shown in Figure 2, amino acid *A *has rank 1, *L *has rank 2 and *C *has rank 20. It can be seen from this figure that the ranks of corresponding unigrams are different in other organisms, but rare-unigrams in one organism are rare overall in all organisms.

**Figure 3:**
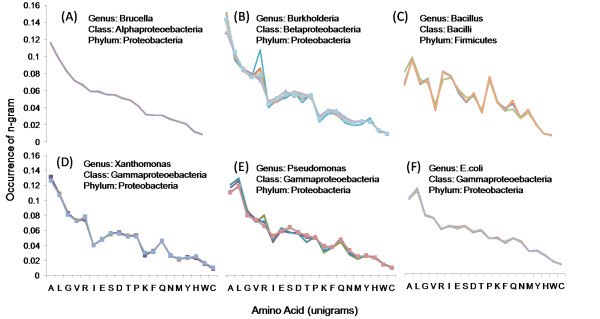
**Unigram distribution in the proteomes of different genera**. Unigram distribution of species from the genera (A) *Brucella*, (B) *Burkholderia*, (C) *Bacillus*, (D) *Xanthomonas*, (E) *Pseudonomas *and (F) *Escherichia *are shown. Within a specific genus, and to some extent within the same class, most species show a similar unigram distribution.

We explored whether this type of analysis would enable classification and groupings of organisms based on similarities in unigram counts and whether unigram preferences are conserved across different species of a given genus. In Figure 3, the unigram distribution is shown separately for six different *genera *(*Brucella*, *Burkholderia*, *Bacillus*, *Xanthomonas, Pseudonomas*, and *E. coli*). To allow comparison across the six plots shown in the figure, the ordering of unigrams along the x-axis is kept consistent and it is that of their rank in *B. suis 1330*. Supplementary material Additional File 1 shows a list of species in each genus. Within a specific genus (i.e., within each subplot) the species show a similar unigram distribution, thereby suggesting that the unigram distribution serves as a *genus signature*. When we compare unigram distributions of different genera within the same *class *(a class is composed of several genera), we find that unigram signatures are similar but not identical for different genera within the same class. The signatures are different when genera are of different classes. For example, in Figure 3, *E. coli*, *Xanthromonas *and *Pseudonomas *belong to the *Gammaproteobacteria *class and show more similar unigram distribution pattern compared to other genera which belong to different classes such as *Brucella *(belongs to *Alphaproteobacteria*), *Burkholderia *(belongs to *Betaproteobacteria*), and *Bacillus *(belongs to *Bacilli*). More examples can be seen in supplementary material Additional File 2.

**Figure 4:**
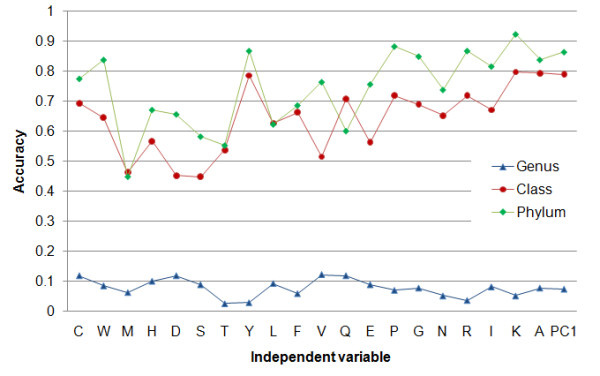
Accuracy of multinomial logistic regression model built with unigram (amino acid) frequencies and first principle component of all unigrams

We also carried out multinomial logistic regression analysis to see whether the whole proteome unigram occurrences can be used to predict genus, class and phylum categories of microbial organisms. A subset of the dataset consisting of genera which have at least 9 species each are used to build the multinomial logistic regression model. Each model is built on the occurrence a single unigram. A 10-fold cross-validation has been carried out for prediction of genus, class and phylum levels. The performance of the model was evaluated by averaging the accuracies over 10 sets. Additionally, dimensionality reduction has been carried out on the on the dataset to explore the prediction power principle components. Figure 4 shows the prediction results of the models built with a single variable (one of the unigram frequencies, or the first principle component). It is seen that the model distinguishes species at class and phylum levels with more than 70% accuracy

### Rare n-grams

While there is a striking variation in rank of certain n-grams in different organisms, n-grams in one organism are usually rare in all organisms. This was observed by [17] and explained by Poddar et al.'s [41] analysis of unigram distributions of various proteomes that the amino acids which are coded by multiple codons occur more frequently than those coded by fewer codons. In the standard genetic code, even among those amino acids that are coded by only one codon, the occurrence of tryptophan (W) was less frequent than the occurrence of methionine (M). This could be linked to the fact that its codon (TGG), when changed the third position becomes a stop codon (TGA), and this would be detrimental to the protein and therefore is usually not chosen by organisms during evolution. Similarly, among those amino acids that are coded by only two codons, the occurrence of cysteine (C) was fewer. The change in the third position of C also leads to a stop codon. Tryptophan and cysteine are the least frequently occurring amino acids of all the proteomes of micro organisms implies that they are not incorporated in proteins unless they play a specific role. Our findings with a larger dataset further support Poddar et al.'s arguments described above.

### Higher order n-gram analysis

**Figure 5:**
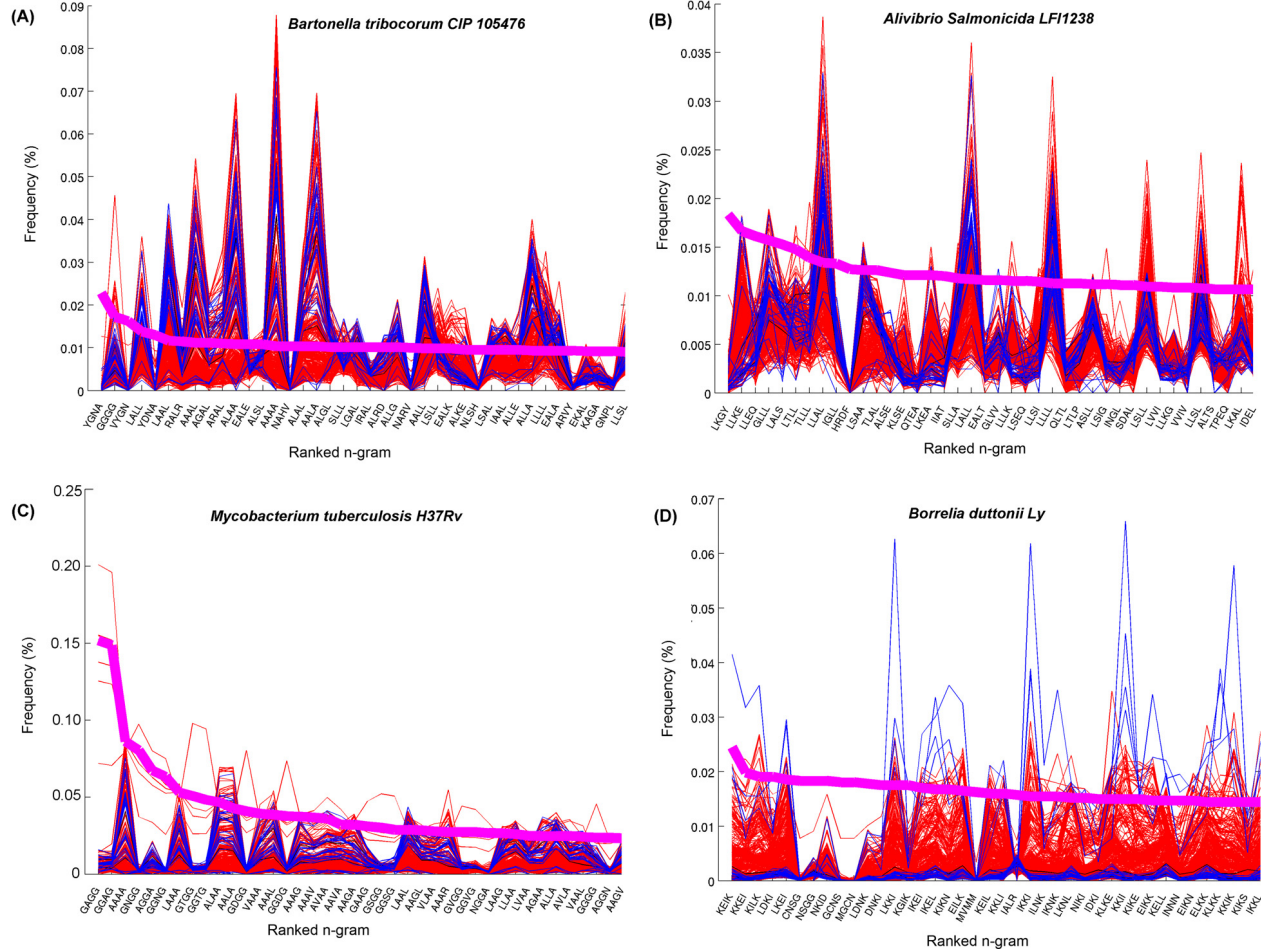
**Comparative Zipf-like analysis for 4-grams**. Top 40 most frequently used 4-grams in (A) *Bartonella tribocorum CIP 105476*, (B) *Alibrio salmonicida LFI1238*, (C) *Mycoplasma tuberculosis H37Ra*, (D, *Borrelia duttoni Ly*. Line colors as in Figure 2. For this larger n, organisms begin to show signature n-grams that occur frequently within their proteome but rarely occurring in other organisms.

As we move to the larger n-grams for Zipf-like analysis, organisms show much more marked differences with some peculiar outliers. Strikingly, we found n-grams that are very frequent in some organisms, yet rare (or completely absent) in others. Examples are shown in Figure 5 for n = 4 in *Bartonella tribocorum CIP 105476 *(Figure 5A), *Alibrio salmonicida LFI1238 *(Figure 5B), *Mycobacterium tuberculosis H37Ra *(Figure 4C), *Borrelia duttoni Ly *(Figure 5D). For example, Figure 5A shows the 4-gram frequencies with *Bartonella tribocorum CIP 105476 *as reference organism. The 4-grams YGNA, YDNA, NAHV, NARV, NLSH, ARVY and GNPL are the top forty most frequent 4-grams in *B. tribocorum*, but are very rare in other organisms. Similarly, exceptionally frequent 4-grams are also found in other organisms (see Figure 5). More examples are shown in Additional File 3.

**Figure 6:**
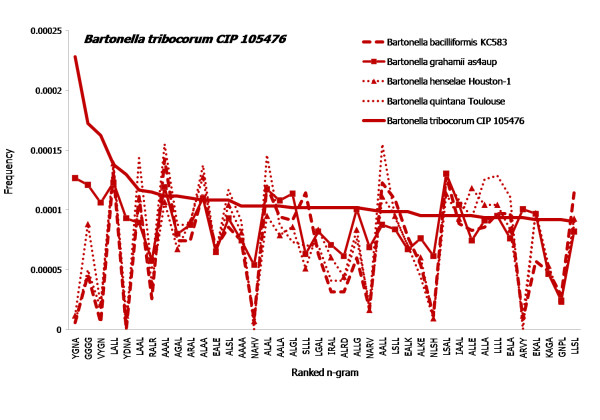
**Comparative Zipf-like analysis for 4-grams within specific genus**. Top 40 most-frequently used 4-grams in *Bartonella tribocorum CIP 105476 *(shown as bold, red line). The corresponding frequencies of these 4-grams in other species of *Bartonella *genus are shown in thin lines.

N-gram proteome composition might also lay a foundation to explore the biological significance of differences in individual organisms. The species in the *Bartonella genus *are facultative intracellular pathogens infecting humans and other animals. The top forty most frequently used 4-grams in *Bartonella tribocorum*, are used very rarely in other organisms (Figure 5A). These 4-grams are rare even among other members of the same genus (Figure 6). The other organisms shown in Figure 5 are: human-specific pathogen *B. baciliformis *and *B. quintana*, feline-specific *B. henselae*, mouse, vole (human)-specific *B. grahamii *, and the rat-specific *B. tribocorum*, all of which belong to the same genus. When the top forty 4-grams of *B. tribocorum *are compared to the top forty 4-grams of other members of the *Bartonella *genus, *B. grahamii *shows most similar pattern for those peculiar 4-grams. The phylogenic tree analysis of *Bartonella *shows *B. tribocorum *and *B. grahamii *are closer to each other than *B. quintana*, *B. henselae*, and *B. baciliformis*. Moreover, *B. tribocorum *and *B. grahamii *have three of the important genomic islands vbh, virB and trw compared to *B. quintana *and *B.henselae *which have just two virB and trw [42]. Moreover, *B. bacilliformas *has none of those islands and has flagellum which makes it different from the other members of *Bartonella*. Even a simple Zipf-like analysis of the top 4-gram distributions of the whole proteome of *B. tribocorum *(Figure 6) can reveal differences among species in a given genus. Using this method can thus reveal proteomic signatures.

**Figure 7:**
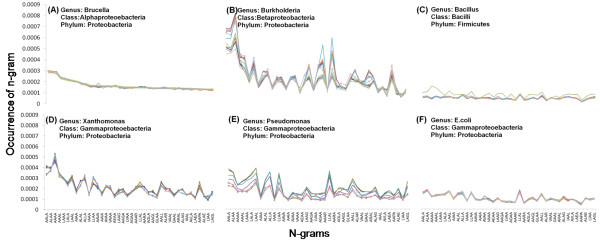
**4-gram distribution in the proteomes of different genera**. Frequency of 4-grams for six *different genera *with the x-axis limited to the top forty most frequently occurring 4-grams of *B. suis*. The six genera shown are (A) *Brucella*, (B) *Burkholderia*, (C) *Bacillus*, (D) *Xanthomonas*, (E) *Pseudonomas *and (F) *Escherichia*.

**Figure 8:**
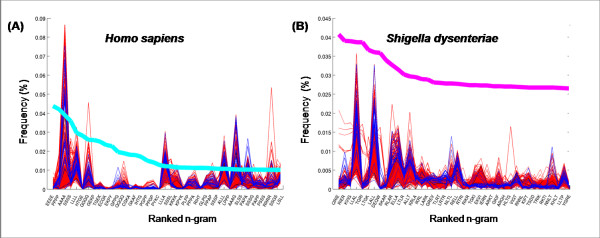
**Usage of very distinct n-gram language models by some organisms**. Top 40 most frequently used 4-grams in (A) *Homo sapiens *(shown as bold, cyan line), (B) *Shigella dysenteria *(shown as bold, magenta line). The corresponding frequencies of 4-grams in other microbes are shown in thin red for animal pathogens and blue for plant pathogens.

We also explored whether higher order n-gram distributions show comparable trend as unigrams, displaying genus specific signatures. Figure 7 shows frequency of 4-grams for six different *genera *(*Brucella*, *Burkholderia*, *Bacillus*, *Xanthomonas, Pseudonomas, E. coli*). The x-axis shows top 40 4-grams of *B. suis 1330 *in all the 6 subplots in Figure 7, to enable cross comparison. The list of species in each genus is given in Additional File 1. Within a specific genus, most species show a similar 4-gram distribution for these particular n-grams, thereby suggesting that the specific 4-gram distribution is conserved within the genus. When we moved the analysis to *class*-level, we observed variation for 4-gram distribution from one genera to the other. Table 1 shows correlation of top forty 4-gram frequencies between *Brucella suis* and corresponding frequencies of these 4-grams in other species, computed as an average over each genera. Only genera with at least 9 species each are considered. The genus to which *Brucella suis* belongs is shown in first row. It is seen that the correlation of 4-gram frequencies is very high at 0.99 for species of the same genera but it is lower with species in other genera whether within the same class or different class. 4-gram analysis is able to reveal genus level signatures as in unigrams, but unlike in unigram analysis, the differences are more pronounced for different genera within the same class. In previous analysis, Ganapathiraju et al. have reported that the n-gram frequencies in human are very different from those of bacterial and archaeal organisms, presumably due to their evolutionary distance from unicellular organisms [17]. In the current analysis over a larger dataset, we find that this is also the case for some prokaryotes such as *Shigella dysentria *as shown in Figure 8. None of the top forty 4-grams of *S. dysentria *are seen with that high frequency in other organisms in the dataset. More examples are shown in Additional File 4.

**Table 1:** Correlation coefficient of 4-gram frequencies across species.

Genus	Correlation Coefficient	Standard Deviation
**1**	**0.99**	**0.0047**

2	0.86	0.0016

3	0.74	0.0415

4	0.67	0.0007

5	0.60	0.0205

6	0.59	0.0349

7	0.56	0.0334

8	0.52	0.2099

9	0.44	0.0832

10	0.38	0.0088

11	0.34	0.0509

12	0.34	0.1105

13	0.33	0.2081

14	0.20	0.1117

15	0.08	0.0725

16	0.00	0.0589

Correlation coefficient of top forty 4-gram frequencies between *Brucella suis *and corresponding 4-gram frequencies in other species, computed as an average over each genera. Only genera with at least 9 species each are considered. Standard deviation also is shown. Brucella belongs to genus 1 (first row) and as seen, the correlation of 4-gram frequencies is very high at 0.99 in comparison to species of the same genera but it is lower with species in other genera whether within the same class or different class.

### Host-specificity

Next, we grouped the microbes by their pathogenecity as animal-infecting or plant-infecting, and compared their n-gram distributions. However, we did not observe significant difference between these two groups. In Figure 3, most of the pathogens infect animal but some species of *Burkholderia *and *Pseudomonas *also infect plants. Plant pathogens that belong to these genera are shown in square markers. As seen in this figure, plant and animal pathogens do not show large difference in terms of their unigram distribution in a particular genus. This might be due to the fact that microbes share strategies for invading the host, whether plant or animal [43]. Some examples of these strategies could be: utilizing the type III protein secretion machinery to inject effectors into cells, or having some effectors to target defensive signal transduction pathways in host cells, or having a common targeting domain in their secreted proteins to enter host cells.

### Perplexity Analysis

The average perplexity of generating a sequence based on the n-gram model of another sequence (cross-perplexity) will tell whether the two are similar to each other in terms of amino acid composition. The average perplexity of a test sequence is larger if the test sequence is dissimilar to the reference sequence. In this study, we investigate whether whole proteome cross-perplexity values are comparable among the same group of microbes. Perplexity models have been computed for many microbial proteomes and tested against all 970 microbial proteomes. Below is one example.

**Figure 9:**
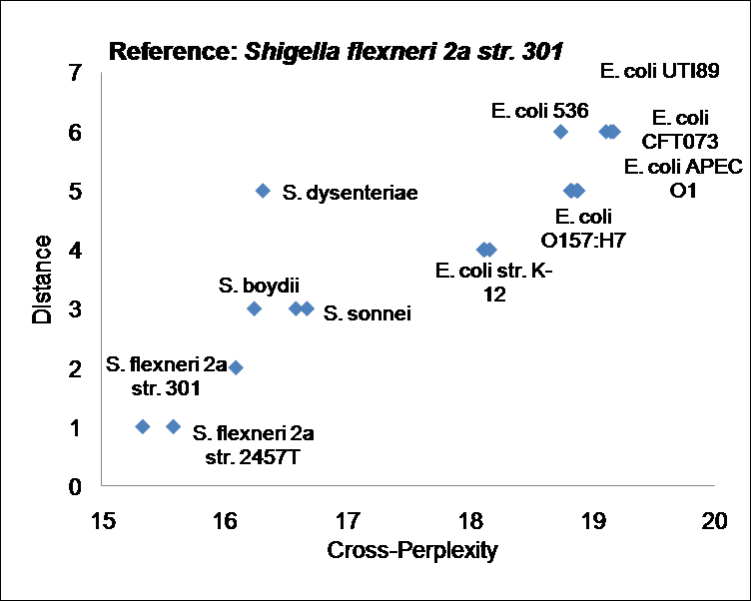
**Cross-perplexity versus branching distance in evolutionary tree within the same or related genus**. Figure shows the cross-perplexity values of organisms that belong to *Shigellae *and *E. coli *genera versus their branching distance in the evolutionary tree [44]. *S. flexneri 2a str. 301 *was used as the reference organism and a language model of 4-grams was trained.

**Figure 10:**
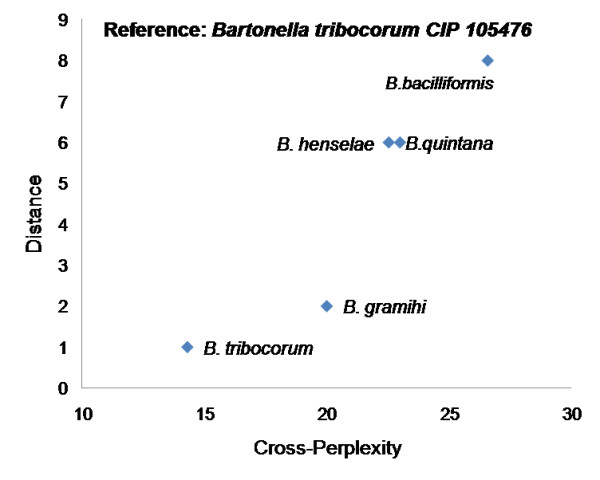
**Cross-perplexity versus branching distance in evolutionary tree within the same genus (another example)**. Figure shows the cross perplexity values of organisms that belong to *Bartonella *genus versus their branching distance in the evolutionary tree. *Bartonella tribocorum CIP 105476 *was used as the reference organism and a language model of 4-grams was trained.

A 4-gram model from proteins of *Shigella flexneri 2a str. 301*, which belongs to the *Gammaproteobacteria *class, was trained. For reference organism self-perplexity (i.e., when test sequence is same as the reference sequence) a perplexity of 15.34 is observed. For the other 969 organisms, the cross-perplexity ranged from 15.59 to 29.5. Figure 9 shows the cross-perplexity values of only the organisms that belong to *Shigellae *and *E. coli *genera are shown with respect to their branching distance from the reference organism. It may be observed that cross-perplexity is proportional to evolutionary distance. The species of *E. coli *also has very close perplexity values with *Shigellae*, consistent with the fact that the species of *Shigellae *are pathotypes of *E. coli*. Similar trend of cross-perplexity being proportional to branching distance is observed in the *Bartonella *genus [42] (see Figure 10), suggesting that the n-gram statistical language model is indicative of evolutionary divergence within a genus.

**Figure 11:**
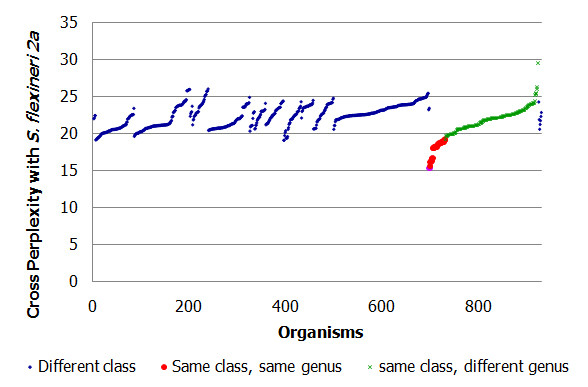
**Cross-perplexity versus branching distance in evolutionary tree across genera for all the 970 organisms**. Figure shows the cross perplexity values with a 4-gram language model for all microbial organisms in the dataset, with *S. flexneri 2a str. 301 *as the reference organism (same as Figure 8). The self-perplexity observed is 15.34 (magenta marker at the bottom end of the red markers).

Next, we extended the comparison of cross-perplexity to test-organisms outside of the genus of the reference organism (see Figure 11). We find that the cross perplexity of organisms within the same (or related genus such as *E. coli*) (red markers in Figure 11) is lower than that for the other organisms. For all 'other' genera within the same class (green markers) as well as for genera of other classes (blue makers), perplexity is higher (ranging from values 20 to 29 in Figure 11). The range of cross-perplexity is not different for genera of the same class compared to genera of other classes although within the same genus as that of the reference organism the cross-perplexity is indicative of evoluationary distance (i.e., the range of blue and green markers is the same in Figure 11), except for its own genus (red markers). For example, *Candidatus carsonella ruddii PV *which belongs to the same class *Gammaproteobacteria *has the highest cross-perplexity 29.5. This microbe has low G+C content whereas the reference organism has a high G+C content.

## Conclusions

The ability to carry out large scale proteome analysis and cross-comparisons across proteomes leads to useful insights in biology, most prominent of them being evolutionary relations. Our analysis illustrates that unigram distribution of amino acids shows a fine resolution signature at the *genus *level (genus signature). We also demonstrated that genus level signatures are similar to each other within a given *class*. Biological language modeling for 970 microbial organisms illustrates significant preferences for particular combinations of amino acids thus strengthening the previous argument that different organisms use different vocabulary. An average cross-perplexity measure is shown to be proportional to evolutionary branch distance within a genus.

Further analysis of microbial genomes in comparison to the biological language models of their host organisms such as human, cow, mouse and plant may reveal further interesting observations.

## Authors' contributions

HUO carried out the computations and analysis under the supervision of MKG. Manuscript is prepared by HUO and MKG. Both authors read and approved the final manuscript.

## Supplementary Material

Additional file 1 **List of species studied in each genus in Figure **2. Click here for file

Additional file 2 Additional figures of unigram distribution of proteomes in Proteobacteria, Firmicutes, Actinobacteria phyla Click here for file

Additional file 3 Additional figures of 4-gram distribution of proteomes in Proteobacteria, Firmicutes, Actinobacteria phyla Click here for file

Additional file 4 **Additional figures for other organisms for the same analysis as Figure **8 Click here for file
