# Supplementary material for: N-gram analysis of 970 microbial organisms reveals presence of biological language models
Source: BMC Bioinformatics. 2011 Jan 10;12:12. doi: 10.1186/1471-2105-12-12 (PMC3027111; doi:10.1186/1471-2105-12-12)
Supplement: Additional file 1 — List of species studied in each genus in Figure 2. [file 1471-2105-12-12-S1.DOC]

**Table 1.1 – List of species studies** in each genus

|  | Species List |
| --- | --- |
| Figure 3A, 6A | *Brucella suis 1330, Brucella abortus S19, Brucella abortus bv. 1 str. 9-941, Brucella canis ATCC 23365, Brucella melitensis ATCC 23457, Brucella melitensis biovar Abortus 2308, Brucella melitensis, Brucella microti CCM 4915, Brucella ovis, Brucella suis 1330, Brucella suis ATCC 23445* |
| Figure 3B, 6B | *Burkholderia ambifaria MC40-6, Burkholderia cenocepacia AU 1054, Burkholderia cenocepacia J2315, Burkholderia cenocepacia MC0-3, Burkholderia mallei ATCC 23344, Burkholderia mallei NCTC 10229, Burkholderia mallei NCTC 1024, Burkholderia mallei SAVP, Burkholderia multivorans ATCC 17616 JGI, Burkholderia pseudomallei 1106a, Burkholderia pseudomallei 1710b ,Burkholderia pseudomallei 668,Burkholderia pseudomallei K96243,Burkholderia 383,Burkholderia vietnamiensis G4 ,Burkholderia xenovorans LB400, Burkholderia glumae BGR1* |
| Figure 3C, 6C | *Bacillus anthracis Ames 0581, Bacillus anthracis A0248, Bacillus anthracis Ames, Bacillus anthracis str. Sterne, Bacillus cereus 03BB102, Bacillus cereus AH187, Bacillus cereus AH820, Bacillus cereus ATCC 10987, Bacillus cereus ATCC14579, Bacillus cereus B4264, Bacillus cereus ZK, Bacillus cereus G9842, Bacillus cereus cytotoxis NVH 391-98, Bacillus licheniformis DSM 13, Bacillus licheniformis ATCC 14580, Bacillus thuringiensis konkukian, Bacillus thuringiensis Al Hakam, Bacillus weihenstephanensis KBAB4* |
| Figure 3D, 6D | *Xanthomonas campestris 8004, Xanthomonas campestris ATCC 33913, Xanthomonas campestris vesicatoria 85-10, Xanthomonas citri, Xanthomonas oryzae KACC10331, Xanthomonas oryzae MAFF 311018, Xanthomonas oryzae PXO99A* |
| Figure 3E, 6E | *Pseudomonas aeruginosa, Pseudomonas aeruginosa LESB58, Pseudomonas aeruginosa PA7, Pseudomonas aeruginosa UCBPP-PA14, Pseudomonas entomophila L48, Pseudomonas mendocina ymp, Pseudomonas syringae phaseolicola 1448A, Pseudomonas syringae pv B728a, Pseudomonas syringae tomato DC3000* |
| Figure 3F, 6F | *Escherichia coli 536, Escherichia coli 55989, Escherichia coli APEC O1, Escherichia coli CFT073, Escherichia coli E24377A, Escherichia coli ED1a, Escherichia coli IAI39, Escherichia coli 0127 H6 E2348 69, Escherichia coli O157H7 EDL933, Escherichia coli O157 H7 EC4115, Escherichia coli O157H7, Escherichia coli O157 H7 TW14359, Escherichia coli S88, Escherichia coli UMN026, Escherichia coli UTI89, Escherichia fergusonii ATCC 35469* |
