# Supplementary figures and images for: N-gram analysis of 970 microbial organisms reveals presence of biological language models
Source: BMC Bioinformatics. 2011 Jan 10;12:12. doi: 10.1186/1471-2105-12-12 (PMC3027111; doi:10.1186/1471-2105-12-12)

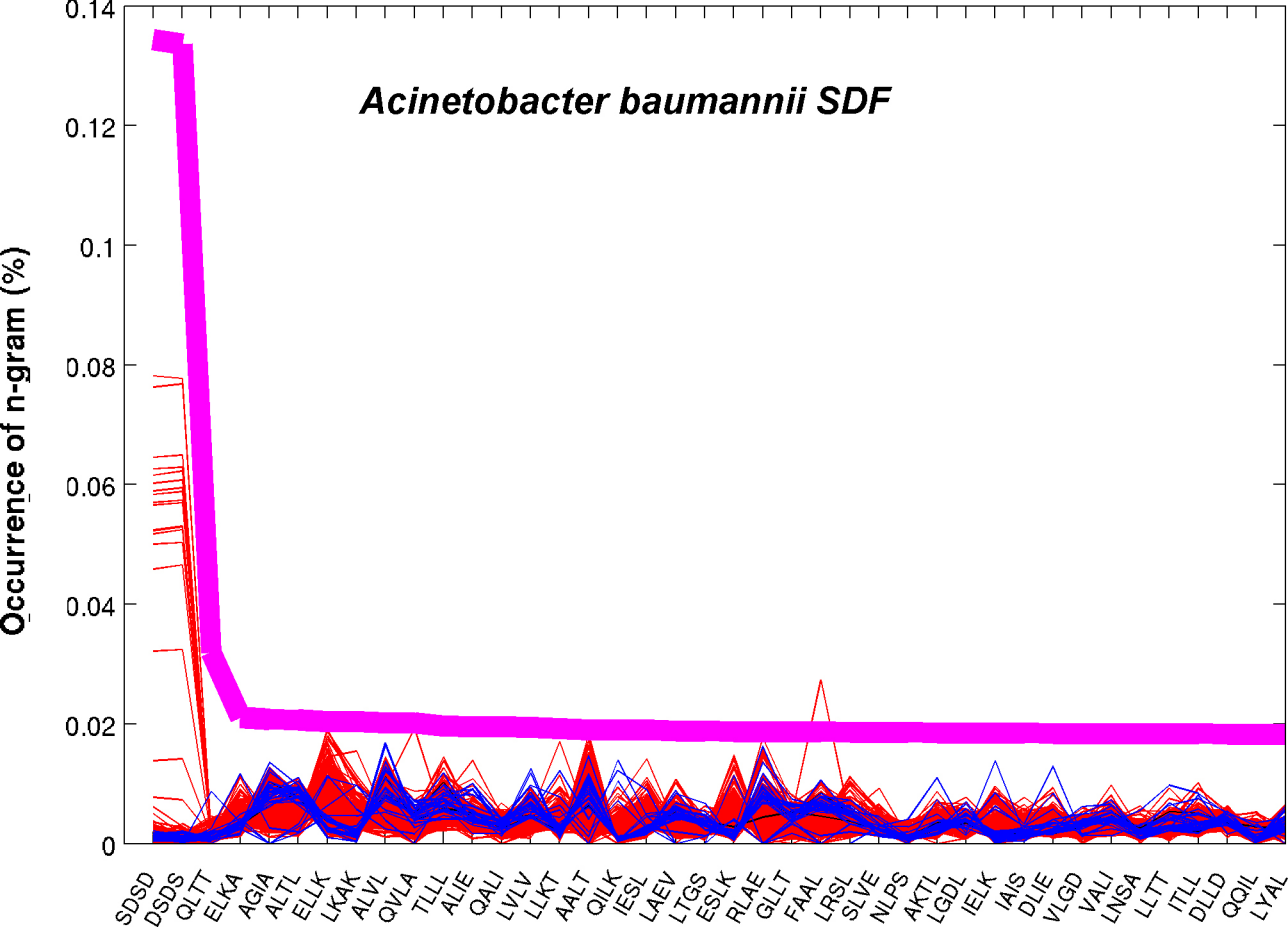

***Aster yellows witches-broom phytoplasma AYWB***

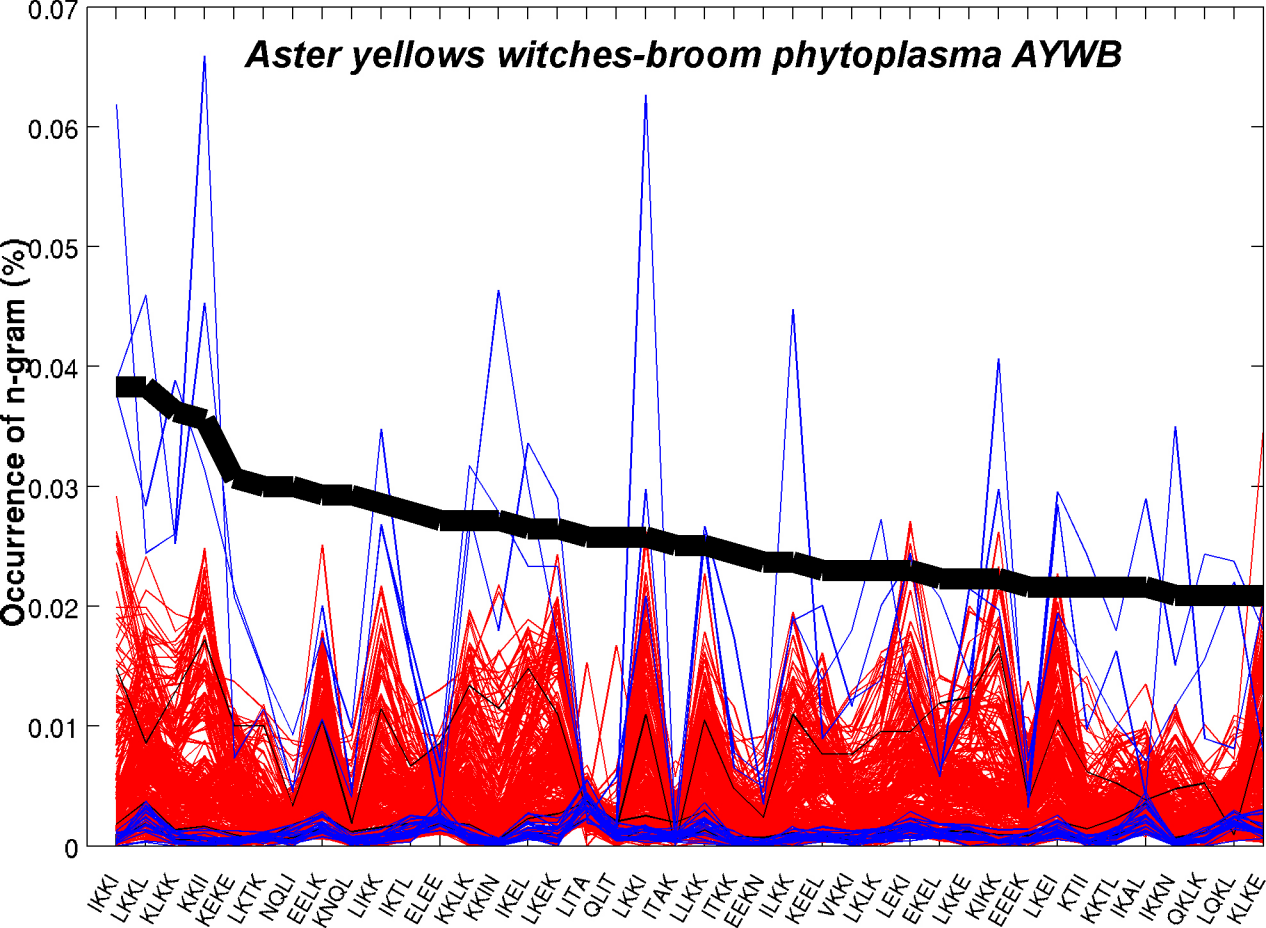

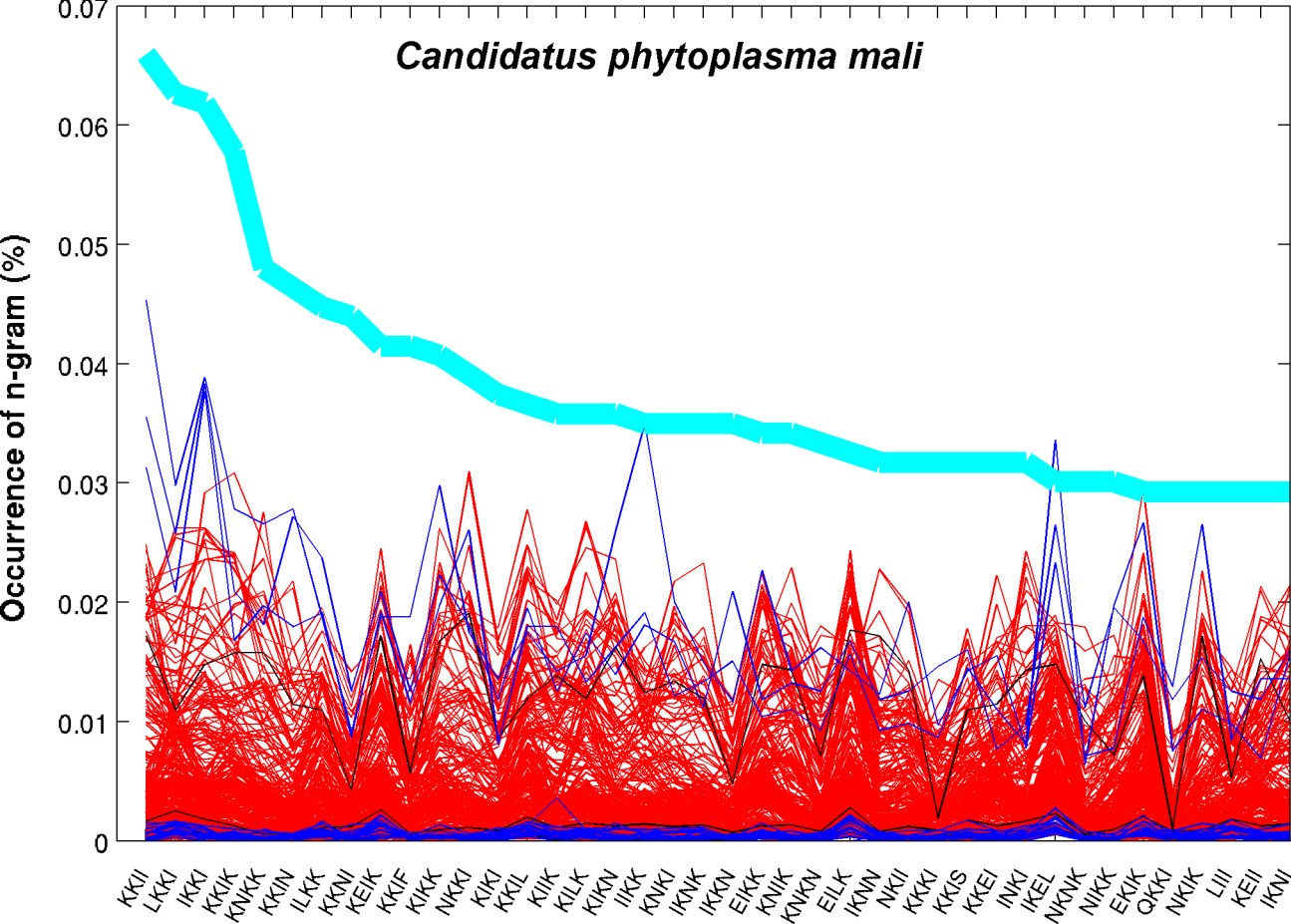

# *Mycoplasma synovia 53*

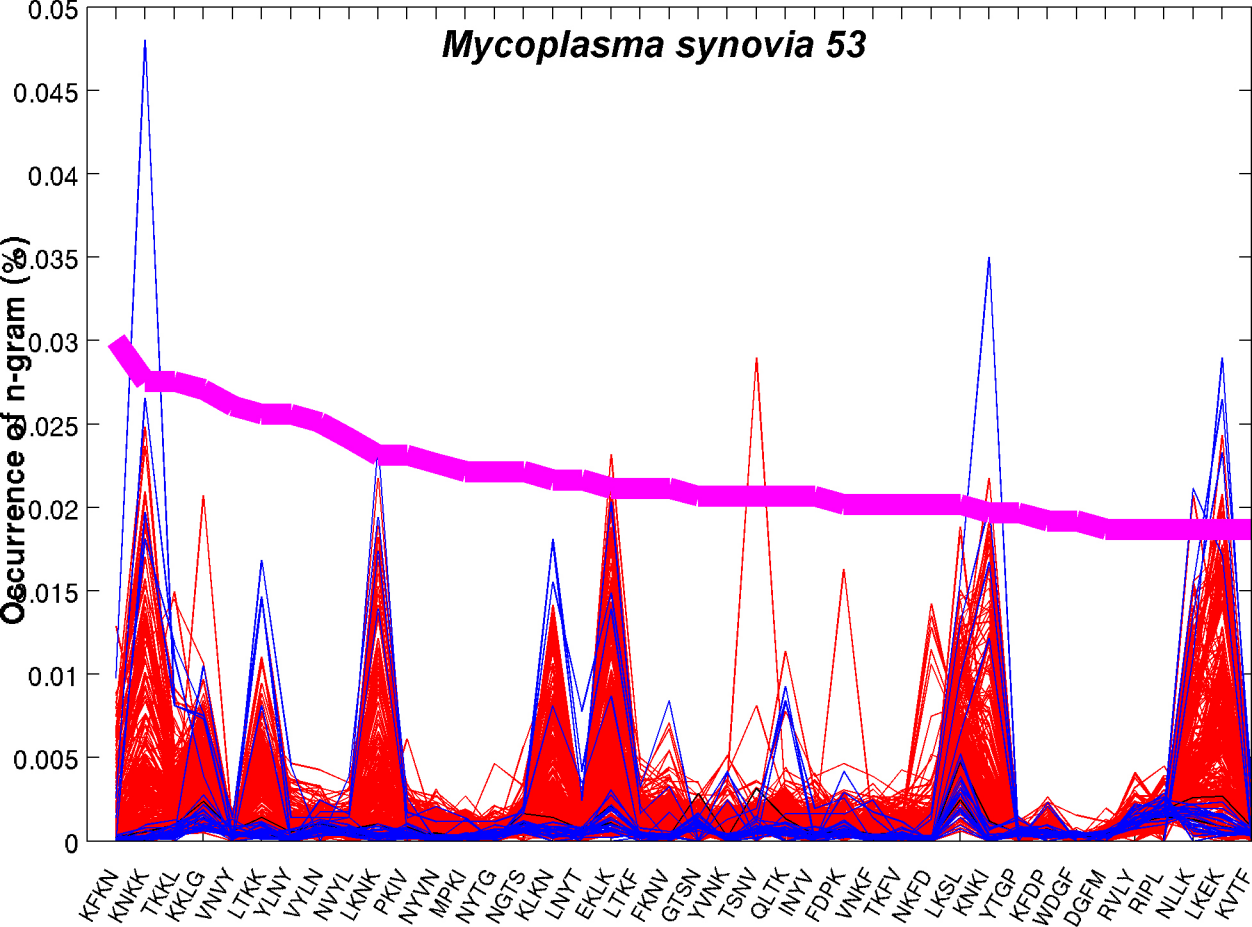

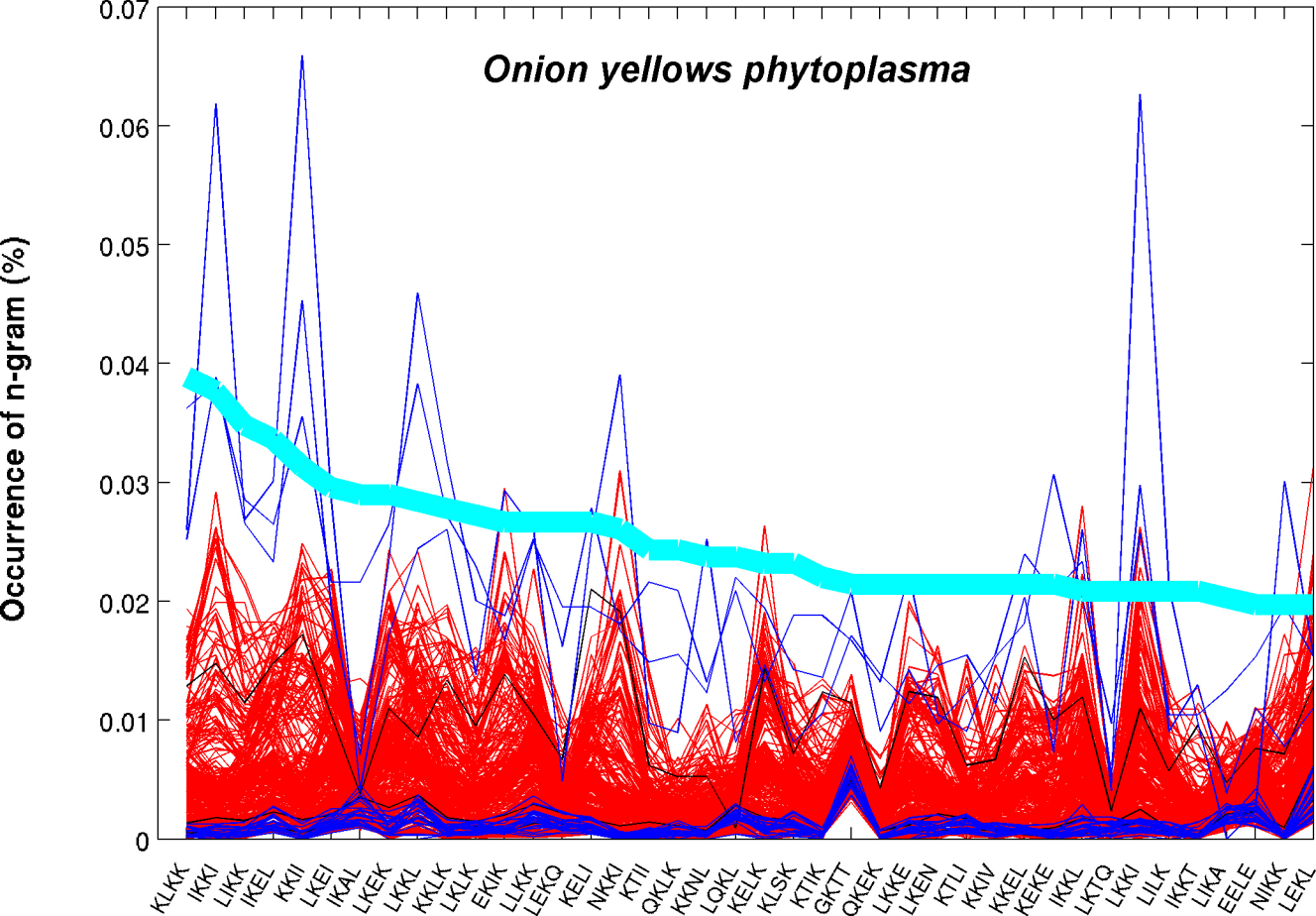

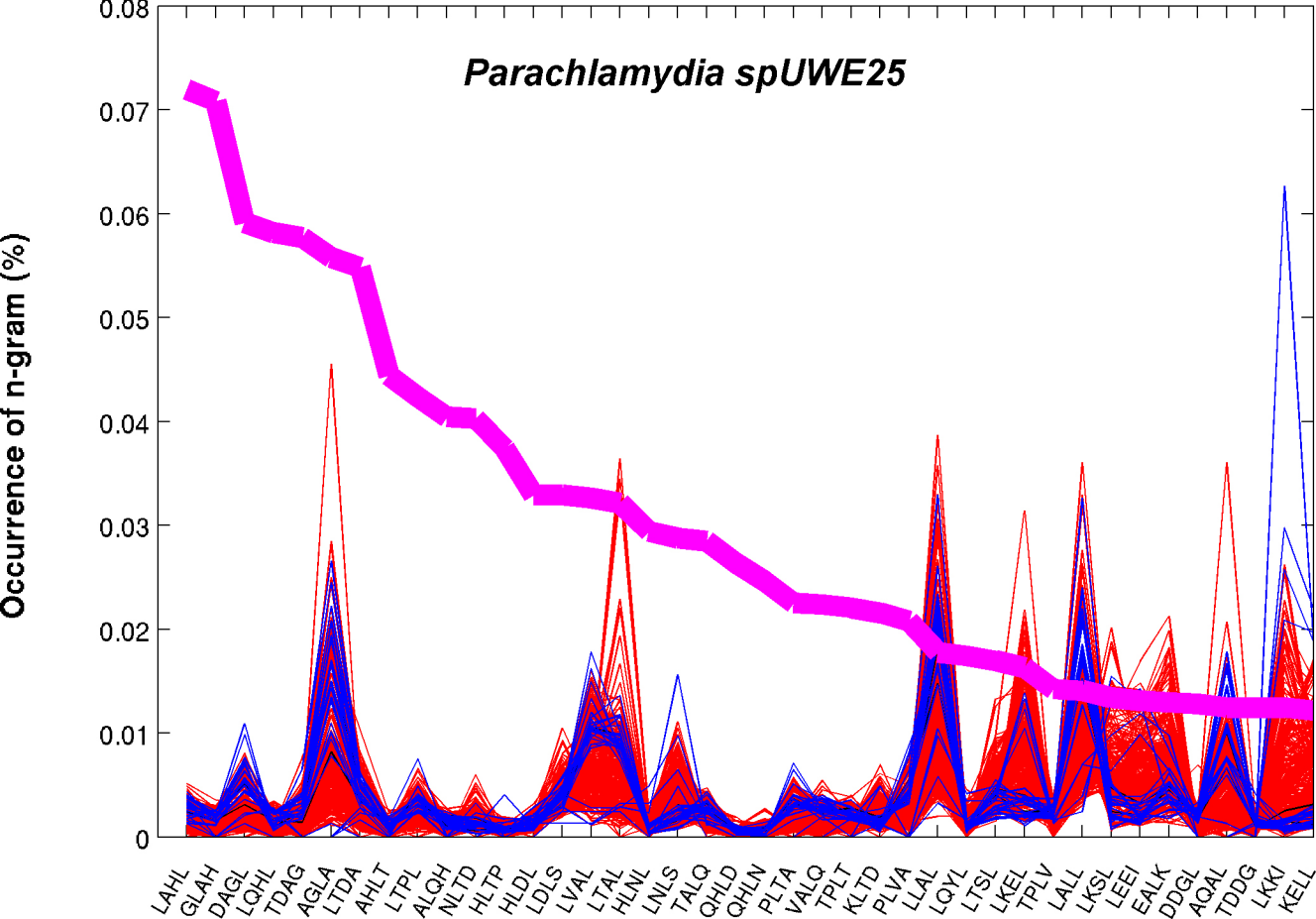

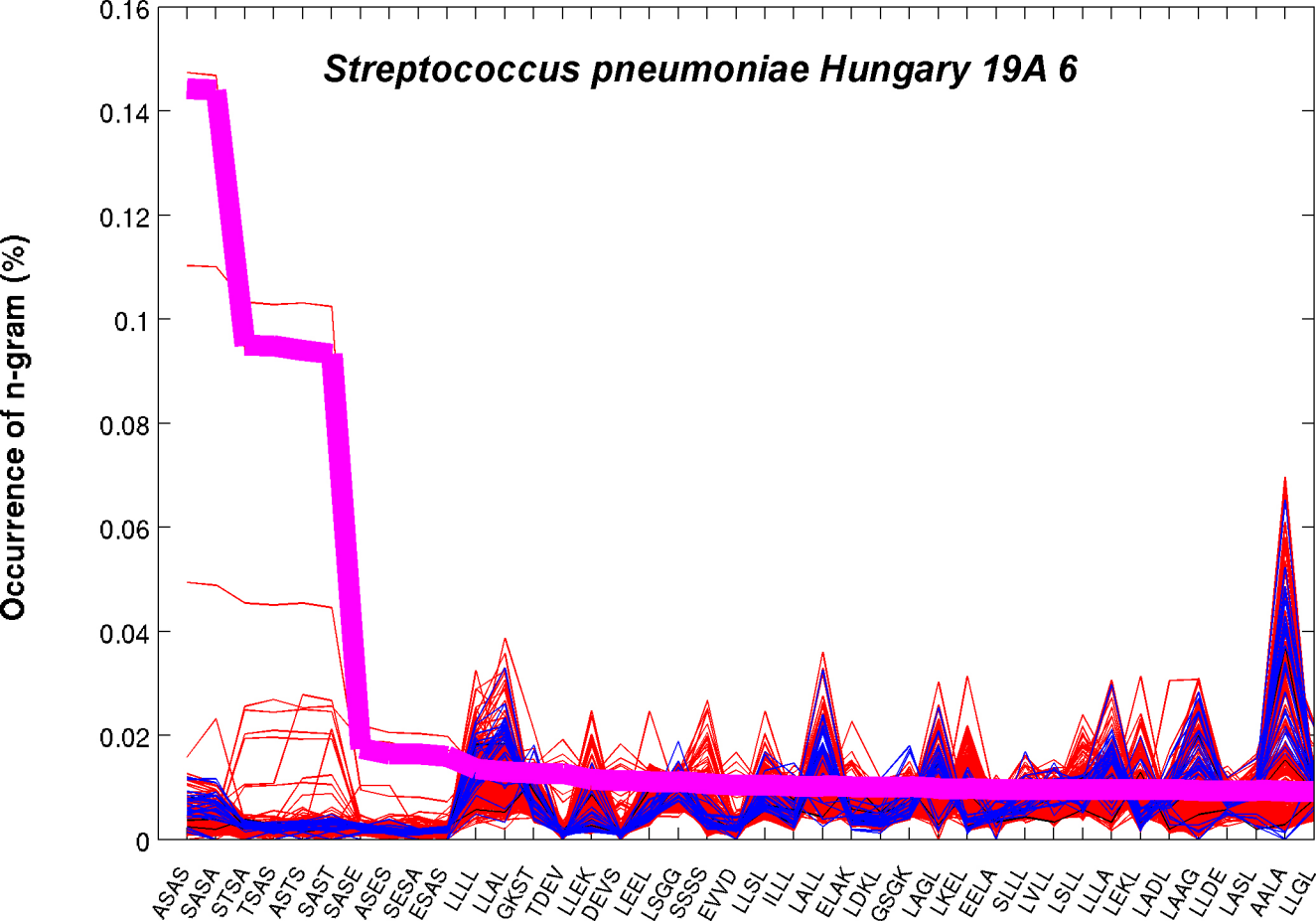

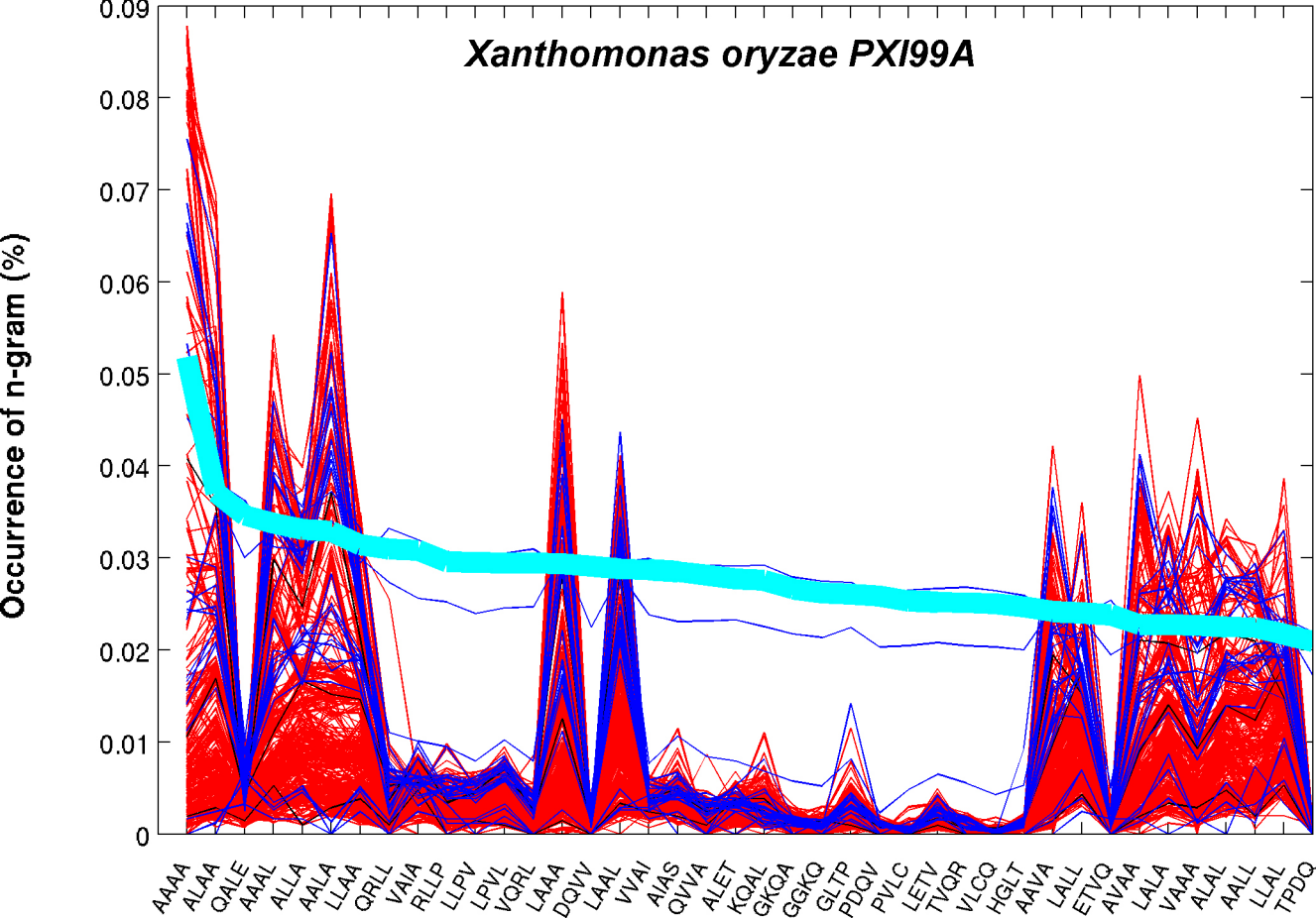

Supplement: Additional file 4 — Additional figures for other organisms for the same analysis as Figure 8 [file 1471-2105-12-12-S4.PDF]
